# Learning and Overnight Retention in Declarative Memory in Specific Language Impairment

**DOI:** 10.1371/journal.pone.0169474

**Published:** 2017-01-03

**Authors:** Ágnes Lukács, Ferenc Kemény, Jarrad A. G. Lum, Michael T. Ullman

**Affiliations:** 1 Department of Cognitive Science, Budapest University of Technology and Economics, Budapest, Hungary; 2 Institute of Psychology, University of Graz, Graz, Austria; 3 Cognitive Neuroscience Unit, School of Psychology, Deakin University, Melbourne, VIC, Australia; 4 Brain and Language Lab, Department of Neuroscience, Georgetown University, Washington, DC, United States of America; Waseda University, JAPAN

## Abstract

We examined learning and retention in nonverbal and verbal declarative memory in Hungarian children with (*n* = 21) and without (*n* = 21) SLI. Recognition memory was tested both 10 minutes and one day after encoding. On nonverbal items, only the children with SLI improved overnight, with no resulting group differences in performance. In the verbal domain, the children with SLI consistently showed worse performance than the typically-developing children, but the two groups showed similar overnight changes. The findings suggest the possibility of spared or even enhanced declarative memory consolidation in SLI.

## Introduction

An increasing awareness of the importance of memory systems in language has inspired researchers to examine these systems in Specific Language Impairment (SLI). Much of the memory research in SLI has focused on working memory, suggesting impairments in this domain, in particular of verbal working memory [1–4]. More recently however, research has begun to examine long-term memory systems, in particular procedural and declarative memory. In this spirit, the Procedural Deficit Hypothesis (PDH) posits that the pattern of language and other deficits in SLI can be largely explained by abnormalities of brain structures underlying procedural memory, in particular frontal and basal ganglia structures [5,6]. Crucially, the PDH also proposes that declarative memory generally remains relatively spared or even enhanced in the disorder, and that it plays important compensatory roles for grammatical and other impairments [6,7]. Although an increasing number of studies of SLI have focused on procedural memory [8], declarative memory has received much less attention.

### Declarative memory

The declarative memory system is rooted in the hippocampus and other medial temporal lobe structures [9–12]. The system underlies the learning, consolidation (the stabilization of memories after their initial acquisition), storage, and use of multiple types of knowledge, including of facts (semantic knowledge), events (episodic knowledge), and words (lexical knowledge). Learning in declarative memory can be very fast, and can occur after as little as a single presentation of the stimulus. The system underlies not only explicit knowledge, that is, available to conscious awareness, but also implicit knowledge [10,13,14]. The functional neuroanatomy of the system has been quite well studied. Briefly, the hippocampus and other medial temporal lobe structures are critical for learning and consolidating new knowledge that depends on this system, though ultimately the long-term storage of this knowledge depends largely on neocortical regions, particularly in the temporal lobes [9–12,15]. Note that the declarative memory systems refers here to the entire neurocognitive system involved in the learning, consolidation, representation, and use of the relevant knowledge, not just to those portions underlying learning and consolidating new knowledge, which is how some researchers refer to the system.

### Declarative memory in SLI

Procedural memory has been increasingly well studied in SLI, with numerous studies now suggesting deficits in this domain, including in consolidation [2,16–20]. For a recent meta-analysis of procedural memory in SLI, which reveals clear impairments in this domain, see Lum, Conti-Ramsden, Morgan and Ullman [8]. However, we still know very little about the status of declarative memory in the disorder.

In the nonverbal domain, studies testing declarative memory have generally revealed largely intact performance in SLI. No differences have been reported between SLI and typically developing (TD) groups in a range of tasks probing a variety of types of nonverbal stimuli, including abstract visual and spatial information, faces, and complex nonverbal sounds [2,21–25]. When such tasks have used easily verbalizable items (e.g., pictures of everyday events), SLI deficits have been found in some [2,23] but not other [21,24] studies. Such impairments may be due to the association of these items with language [26]. Type of task also seems to have an influence. For example, Kuppuraj et al. [25] found intact nonverbal declarative memory performance in SLI with incidental encoding and later recognition (similar to the approach employed here), while performance lagged behind controls with intentional encoding and later recall.

In the verbal domain, studies have generally–but not always [21,27,28]–found impairments in tasks probing declarative memory [2,21–24,27–31]. However, most of these studies used list learning tasks, which rely heavily on working memory (due to the repetition of items during the learning phase). Since verbal working memory is often impaired in SLI [1–4,6], any deficits at these tasks could be due to problems with working memory rather of declarative memory itself [2,6]. Indeed, verbal declarative memory deficits observed in a range of tasks in Lum et al. [2] were reduced or eliminated after covarying out working memory (and were completely absent after language abilities were controlled for). More recently, Lum, Ullman, and Conti-Ramsden [32] found that in a list learning task only those children with SLI who had working memory deficits showed impairments at declarative memory; those without working memory problems showed normal performance at the task. Together, the data suggest that declarative memory deficits in SLI may be due largely, if not entirely, to accompanying working memory deficits, as well as to their underlying language impairments.

However, almost all studies of declarative memory in SLI have focused on the initial stages of learning, in which the acquired knowledge is typically tested after a short delay of minutes following encoding (enough time to reduce the likelihood of maintaining the information in working memory). But subsequent *retention* of that knowledge is also critical, since the goal of learning is generally to retain information beyond the range of a few minutes. This is especially the case, of course, with language. To our knowledge, however, there is only one study that specifically examined longer-term retention, i.e., one day or more after initial learning [33]. In this study, word learning was tested in a heterogeneous group of young adults with different forms of language impairment (with previous diagnoses not only of “language impairment”, but also of dyslexia, learning disability, dysnomia, or a combination of these) and was compared to a TD group of the same age. The study was designed to separately examine learning word forms, word referents, and links between form and referent. Retrieval of items was tested immediately after training; after 12 hours (either involving sleep or not); 24 hours after training; and one week later. The main result relevant to retention was that after delays involving sleep the language-impaired group did not differ from the TD group at remembering referents or form-referent links, but did show impairments at retrieving word forms. The authors concluded that the “consolidation of declarative memory is a relative strength for young adults with LI [language impairment]”, though primarily when the items were not purely verbal in nature, perhaps since learning novel word forms may also depend on procedural memory [6]. However, weaknesses of the study involving participant criteria suggest caution in generalizing the results. Moreover, a dearth of direct group comparisons in the paper makes it difficult to interpret the results. Finally, purely nonverbal information was not tested, since even the nonverbal referents were learned initially in the context of the novel words that referred to them. Together with the fact that this is the only study to date to examine retention in SLI suggests that further studies are needed.

### The present study

The aim of the present study was to examine both learning and retention in children with SLI, as compared to TD children, in both nonverbal and verbal domains. The study was designed to minimize the influence of functions that can affect performance on declarative memory tasks, and are often impaired in SLI, namely working memory and free recall–both of which depend on frontal/basal ganglia circuits, which appear to be abnormal in SLI [6,34]. We therefore tested declarative memory with a recognition memory task, following incidental encoding. To test initial learning we probed recognition memory 10 minutes after encoding. Both nonverbal items (pictures of real and novel objects) and verbal items (auditorily presented real and novel words) were examined. To test for retention we probed recognition memory of the same items 24 hours later.

Consistent with the PDH and previous studies, we predicted largely intact recognition memory for nonverbal items in SLI at initial learning (after the short delay of 10 minutes). In contrast, we expected the possibility of impairments at recognition memory for verbal items at this time point. Given the dearth of previous evidence regarding retention in declarative memory in SLI, we had no clear predictions for recognition memory following the overnight delay.

## Materials and Methods

The Budapest University of Technology and Economics Behavioral and Biomedical Institutional Review Board reviewed and approved the study (IRB #: IRB00004964 Project Title: Nonlinguistic abilities in Specific Language Impairment). All children were tested with the informed written consent of their parents (by asking them individually to sign a detailed consent form), in accordance with the principles set out in the Declaration of Helsinki, and approved by the Budapest University of Technology and Economics Behavioral and Biomedical Institutional Review Board. In approving this Research Project, the Review Board followed the requirements of the Common Rule and the Helsinki Agreement.

### Participants

Children with SLI were recruited from two special schools for children with language impairment, which were in and near Budapest, Hungary. The children had been referred to these schools by speech and language therapists working in clinical practice. Recruitment and screening for this study lasted between 2 and 3 months at each school. No eligible children declined participation. All children in the SLI group met the following inclusion and exclusion criteria, which are commonly used in SLI research [1,35]. They scored at least 1.5 standard deviations (*SD*s) below the mean for their age on at least two of the following four language screening tests: (1) a Hungarian version of the Peabody Picture Vocabulary Test [36,37]; (2) the Hungarian version of the Test for the Reception of Grammar [38,39]; (3) the Hungarian Sentence Repetition Test [40]; and (4) a Hungarian nonword repetition test [41]. Their nonverbal IQ as measured by the Raven Progressive Matrices (RPM) was in the normal range, corresponding to a score above 85 IQ points [42]. Their hearing was also assessed as normal. Typically developing children were recruited from two regular schools, which had no special selection processes for children. All TD children scored normally, that is, above 1.5 *SD*s below the mean for their age, on all four language screening tests. The TD children were matched individually (pair-wise) to children with SLI on chronological age and sex; the two groups were also matched group-wise on nonverbal IQ [42]. None of the TD children were known to have been diagnosed with any neurodevelopmental, psychiatric, or neurological disorder. Similarly, none of the children with SLI had any known current or past neurodevelopmental, psychiatric, or neurological disorders other than SLI; e.g., children with comorbid autism or ADHD were excluded. A total of 42 children were tested: 21 with SLI and 21 who were typically developing (TD). Demographic and screening data for the two groups are shown in Table 1.

**Table 1:** Demographic and screening data for the two groups.

	SLI	TD	Group Comparisons	
n	21	21		
Sex	15M, 6F	15M, 6F		
Age (years)	8.89 (1.06)	8.85 (1.03)	*F*(1, 40) = 0.02, *p* = .884	
Vocabulary (PPVT; raw scores)	98.00 (19.60)	124.43 (12.50)	*F*(1, 40) = 27.14, *p* < .001	
Grammar (TROG; blocks raw score)	13.52 (2.02)	18.05 (1.47)	*F*(1, 40) = 69.21, *p* < .001	
Sentence Repetition (raw scores)	20.24 (8.33)	37.67 (2.80)	*F*(1, 40) = 82.60, *p* < .001	
Nonword Repetition (span)	3.29 (1.27)	6.48 (0.98)	*F*(1, 40) = 82.98, *p* < .001	
Nonverbal IQ (RPM; standard score)	103.90 (9.82)	107.38 (11.19)	*F*(1, 40) = 1.14, *p* = .291	

Note. Means (and standard deviations) are shown for each variable. Results from one-way ANOVAs are shown for group differences. SLI: specific language impairment; TD: typically developing; M: male; F: female. Vocabulary scores are computed from the PPVT (Peabody Picture Vocabulary Test), grammar scores from the TROG (Test for the Reception of Grammar), and nonverbal IQ scores from the RPM (Raven’s Progressive Matrices); see main text.

### Procedure

Declarative memory was tested with a recognition memory task developed by the Brain and Language Lab at Georgetown University, and modified as appropriate for the Hungarian participants in the present study. The task assesses encoding, initial learning (recognition after a short delay of 10 minutes) and retention (recognition again after a delay of 1 day) in declarative memory. Separate subtasks examine nonverbal and verbal learning, using two sets of stimuli: the nonverbal subtask visually presents pictures of real and novel objects, while the verbal subtask auditorily presents words and nonwords.

Each of the two subtasks consists of three phases. First, in the Encoding phase, participants are presented with 32 real and 32 novel items; that is, pictures of 32 real and 32 made-up objects in the nonverbal subtask, or 32 real and 32 made-up words in the verbal subtask. Participants are asked to make a real/novel decision on each item, that is, an object decision in the nonverbal subtask, and a lexical decision in the verbal subtask; see below for further details. This incidental encoding task is followed by an initial Recognition phase after a 10-minute delay, and a Retention phase after a 24-hour delay. These two phases are virtually identical. Both phases present all 64 target items that were seen or heard in the encoding phase (old items), together with 64 foils (new items), for a total of 128 items. Half the old items and half the new items are real (objects or words) and half are novel (novel objects or nonwords). The foils (new items) are entirely new in both the Recognition and Retention phases; that is, the foils in the Retention phase are foils that were not presented previously in the Recognition phase.

Stimuli were presented on a Lenovo z61m PC laptop running Windows 7, using E-Prime 1.2 [43]. A display resolution of 1024 x 768 pixels was used. The objects in the nonverbal subtask were presented as 640 x 480 pixel pictures. Children sat approximately 40–60 centimeters from the screen. Testing took place in a quiet room in the children’s school. For the verbal subtask, the stimuli were presented via headphones to further decrease noise. Responses were made on the left and right buttons located just below the touchpad on the laptop (see below for Instructions).

Items in the nonverbal subtask were presented with the following presentation and timing parameters in all three phases (Encoding, Recognition, Retention). A 1000 millisecond (msec) preparation period with a fixation cross at the center of the screen signaled the imminent presentation of each new item; during the first 200 msec of this preparation period a tone was also presented. After this 1000 msec preparation period, the picture appeared in the center of the screen for 500 msec. If the participant responded during this 500 msec presentation period, the item disappeared. Following the disappearance of the picture (at or before 500 msec), the fixation cross reappeared on the screen. If the participant responded prior to 5000 msec after the appearance of the picture on the screen (i.e., during the allowable response period, including during the initial 500 msec), the next item was when the experimenter pressed a mouse button, at which point the 1000 msec preparation period for the next item began. If the participant did not respond within the 5000 msec response period, then at 5000 msec a 1000 msec time-out period occurred (a 400 msec time-out tone, together with a fixation cross which lasted the full 1000 msec; note that the time-out tone had a different frequency from the tone in the preparation period), after which the 1000 msec presentation period for the next item began.

The presentation and timing parameters for the words/nonwords in the verbal subtask were identical to those for the real/novel objects in the nonverbal task except that the presentation duration of the stimulus (word/nonword) was variable (rather than the consistent 500 msec duration in the nonverbal subtask), lasting the duration of the sound file of the item. As in the nonverbal subtask, the response period was 5000 msec from the onset of the stimulus.

The following instruction procedures were followed for both subtasks. All instructions were given in Hungarian. Each of the three phases (Encoding, Recognition, Retention) began with instructions, which were presented on the screen and also read out and explained by the experimenter. Before all three phases, participants were instructed to place their left and right index fingers on the left and right buttons located just below the touchpad on the laptop, and to make a response by pressing one of these buttons. In the Encoding phase, participants were instructed to decide as quickly and accurately as possible whether the object or word was Real or Made-up, and to press one of two buttons accordingly. In both the Recognition and Retention phases, participants were instructed to indicate by button press whether the item they were presented with was or was not previously presented in the Encoding phase (Yes/No decision on whether they had seen or heard the item earlier). Instructions were followed by 3 practice items in the encoding phase, and 6 practice items in both the Recognition phase (3 old, that is, the three practice items from encoding; and 3 new, that is, that were not presented as practice or experimental items during Encoding) and the Retention phase (3 old, the same practice items as in Encoding and Recognition; and 3 new, which had not been presented during either Encoding or Recognition).

For each subtask two versions (A and B) were created. In one version the left button was associated with Real (Encoding) or Yes, presented earlier (Recognition and Retention), and the left button with Made-up/No, while in the other group, the mapping was the reverse. The two versions were alternatively assigned to consecutive participants within each participant group (SLI and TD). During the task, a reminder appeared at the bottom of the screen indicating the mapping of the buttons.

Participants (within each group and version) were randomly assigned to one of two subtask orders. In one order, participants were first given the Encoding phase of the verbal subtask, followed by the Encoding phase of the nonverbal subtask, then the Recognition phase of the verbal subtask, then the Recognition phase of the nonverbal subtask, and 24 hours later the Retention phases of first the verbal then the nonverbal subtask. In the other order subjects were given first the nonverbal and then the verbal subtask for each phase. Participants were given the Encoding and Recognition phases at varying times, between about 9am and 4pm, and were tested on Retention about 24 hours later. Encoding phases took 5–7 minutes to complete, and Recognition and Retention phases took between 9–11 minutes to complete. Participants took a short self-paced break between the two Encoding phases. The delay between each Encoding phase and its corresponding Recognition phase was about 10 minutes.

### Stimuli

The real and made-up object stimuli in the nonverbal task were identical to the stimuli developed in the original version of the task developed in the Brain and Language Lab. These items were validated as real and novel objects by the Hungarian experimenters. The items were black and white line drawings of real and made-up objects. Images of real objects had been taken from various sources, and then modified as necessary. Sources included a number of different clipart galleries (including free websites and purchased collections) and the Snodgrass and Vanderwart [44] set of pictures. Images for made-up objects had been taken from Eals and Silverman [45], Cornelissen et al. [46] and Williams and Tarr [47]; they were then modified as necessary, including to reduce nameability. Low nameability was confirmed in previous pilot studies run in the Brain and Language Lab. All images were resized, touched up, rotated, and/or converted to black-and-white to create the final set of stimuli produced by the Brain and Language Lab. The items were presented in a pseudo-randomized order, with no more than 3 consecutive real or made-up objects.

**Fig 1:**
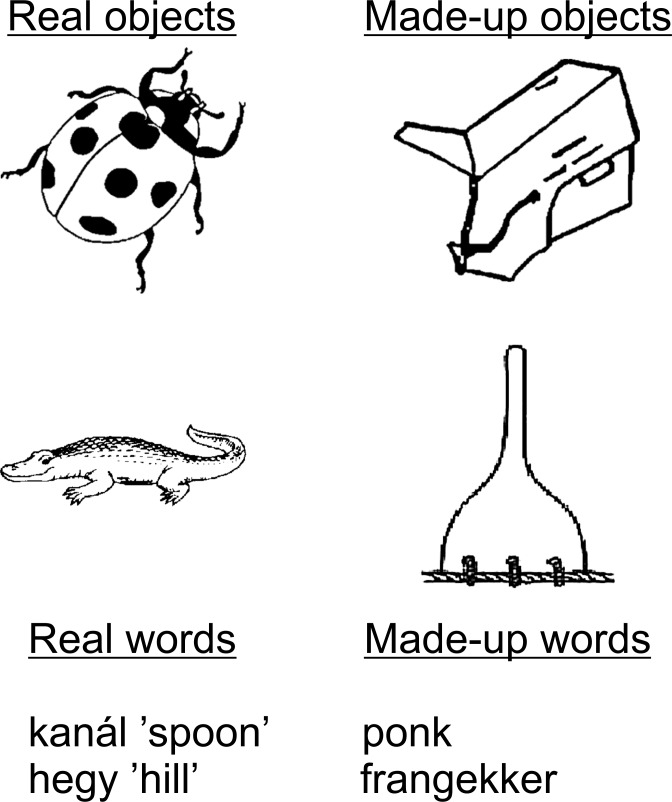
Example stimuli from the nonverbal subtask (real and made-up objects) and the verbal subtask (real and made-up words).

Stimuli in the verbal subtask comprised auditorily presented real words (concrete nouns) and made-up words (Hungarian adaptations of the English nonwords created by the Brain and Language Lab). The nonwords were matched to the real words in phonological length (range: 1–5 syllables) and syllable structure (e.g., a CVCC word was matched as closely as possible to a CVCC nonword) for all three sets of real/made-up words: the target items, the foils presented during Recognition, and the foils presented during Retention. Additionally, these three sets were matched on the same factors. All items conformed to the rules of Hungarian phonotactics. All words and nonwords were digitally recorded by a male native Hungarian speaker in 16 bit, 44100 Hz, 705 kbps mono–not stereo–wave format (though these were later presented to participants to both ears via the headphones). All sound files were edited to reduce their length to the length of the word or nonword. The length of the sound files ranged between 465 and 1311 msec. See Fig 1 for examples of stimuli in both the nonverbal and verbal subtasks.

## Results

Between and within group differences in recognition memory were examined with 2 (Group: SLI vs. TD) X 2 (Real/Novel: Real vs. Novel) X 2 (Delay: 10 minutes/Recognition vs. 24 hours/Retention) mixed ANOVAs run separately on the Nonverbal and Verbal subtasks. In these analyses Group was the between-subject factor, and Real/Novel and Delay were within-subject factors. In order to minimize effects of response bias, accuracy was entered in the analyses as normalized *d'* (*d*-prime) scores (*d’* = z(hit rate)—z(false alarm rate)). For all analyses, we report partial eta-squared (*η*_*p*_^2^) as a measure of effect size. Reaction times were also analyzed, using median RTs (based on raw RTs for correct responses only); however, these analyses are not presented here, since there were no main effects of Group or interactions with Group (all *p*s > 0.1).

### Recognition and retention of nonverbal information

Performance on the Nonverbal subtask is shown in Table 2. Both the SLI and TD groups showed above-chance performance at both Real and Novel items, for both Recognition and Retention (Table 2).

**Table 2:** Recognition and retention accuracy for nonverbal information.

	SLI	TD
Recognition (10 minute delay)		
Real		
*d’*	1.41 (1.12) ***	2.17 (1.05) ***
Hit rate	0.77 (0.20)	0.78 (0.19)
False alarm rate	0.33 (0.32)	0.14 (0.15)
Novel		
*d’*	0.65 (0.75) **	0.92 (0.78) ***
Hit Rate	0.38 (0.23)	0.38 (0.16)
False Alarm Rate	0.21 (0.25)	0.16 (0.17)
Retention (24 hour delay)		
Real		
*d’*	1.90 (1.21) ***	2.18 (0.95) ***
Hit rate	0.74 (0.23)	0.75 (0.22)
False alarm rate	0.20 (0.24)	0.11 (0.10)
Novel		
*d’*	1.12 (0.76) ***	1.12 (0.94) ***
Hit Rate	0.45 (0.23)	0.38 (0.21)
False Alarm Rate	0.15 (0.21)	0.10 (0.11)

Note. Accuracy in the Nonverbal subtask, showing means (and standard deviations) of *d*’, as well as of hit rates and false alarm rates. SLI: specific language impairment; TD: typically developing. Asterisks indicate performance greater than chance (mean *d*’s significantly greater than zero, one-sample t-tests, df = 20):

***: p < .001

**: p < .01

*: p < .05.

**Fig 2:**
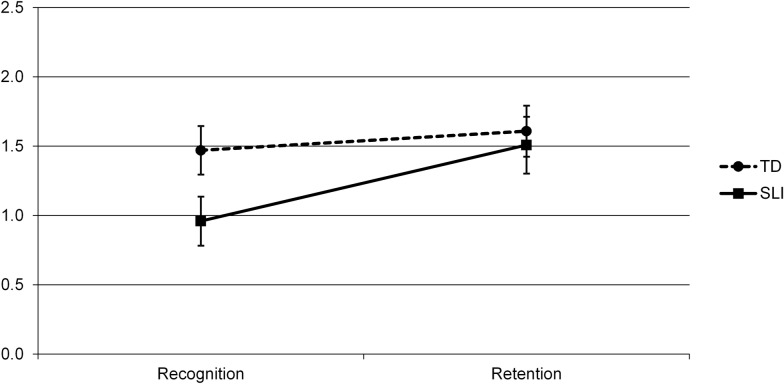
Nonverbal subtask performance by Group (SLI vs. TD) and Delay (10 minutes/Recognition vs. 24 hours/Retention), showing mean *d*’ scores and standard errors.

**Fig 3:**
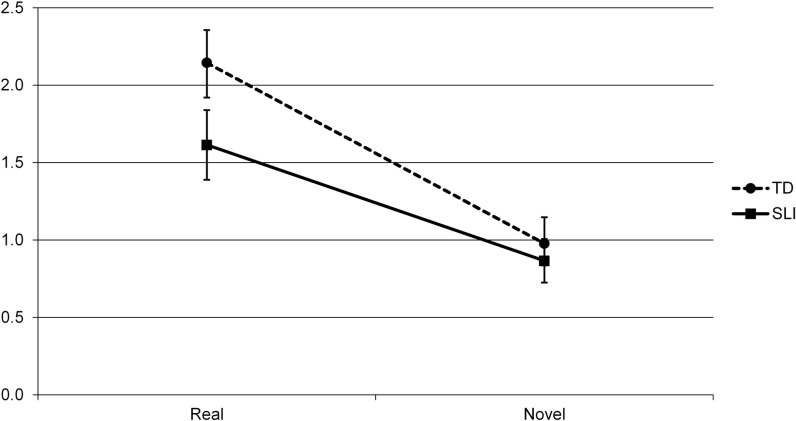
Nonverbal subtask performance by Group (SLI vs. TD) and Real/Novel, showing mean *d*’ scores and standard errors.

The 2 (Group) X 2 (Real/Novel) X 2 (Delay) ANOVA yielded main effects of Delay (*F*(1, 40) = 11.774, *p* = .001, *η*_*p*_^*2*^ = .227), with better performance at Retention than Recognition over both groups, and of Real/Novel (*F*(1, 40) = 95.450, *p* < .0001, *η*_*p*_^*2*^ = .705), with Real objects recognized better than Novel ones. There was no main effect of Group (*F*(1, 40) = 1.63, *p* = .209, *η*_*p*_^*2*^ = .039). However, the two significant main effects were qualified by a significant interaction between Group and Delay (*F*(1, 40) = 1.478, *p* = .034, *η*_*p*_^*2*^ = .107) and an interaction between Group and Real/Novel that approached significance (*F*(1, 40) = 1.575, *p* = .057, *η*_*p*_^*2*^ = .088); see Figs 2 and 3. There were no other interactions (Real/Novel X Delay; *F*(1, 40) = .433, *p* = .514, *η*_*p*_^*2*^ =. 011; Group X Real/Novel X Delay: *F*(1, 40), = .651, *p* = .424, *η*_*p*_^*2*^ = .016).

We first followed up on the Group X Delay interaction, collapsing over both object types (Real and Novel). There was no effect of Delay in the TD group; that is, there was no significant difference in accuracy for the TD children between Recognition and Retention (*F*(1, 20) = 2.004, *p* = .172, *η*_*p*_^*2*^ = .091). However, for the SLI group, accuracy was significantly higher at Retention than Recognition (*F*(1, 20) = 20.340, *p* < .001, *η*_*p*_^*2*^ = .504). Additionally, the SLI group was worse than the TD group at Recognition (*F*(1, 40) = 4.211, *p* = .047), but not at Retention (*F*(1, 40) = .133, *p* = .718). See Fig 2. S1 Fig shows *d*’ performance at Recognition and Retention at the nonverbal subtask for each child with SLI (S1 (A)) and each TD child (S1 (B)).

Next, we followed up on the Group X Real/Novel interaction that approached significance, collapsing over both Delay periods (10 minutes/Recognition and 24 hours/Retention). The effect of Real/Novel was significant in both the TD and SLI groups, with better performance (i.e., collapsed over both Delay periods) on Real than Novel objects in both groups, though this effect was larger for the TD children (TD: *F*(1, 20) = 79.707, *p* < .0001, *η*_*p*_^*2*^ = .799; SLI: *F*(1, 20) = 49.626, *p* < .0001, *η*_*p*_^*2*^ = .713). Additionally, the effect of Group (again, collapsed over both Delay periods) showed a trend for Real objects, with somewhat better performance by the TD than the SLI group (*F*(1, 40) = 2.947, *p* = .094, *η*_*p*_^*2*^ = .069), while it there was no group difference for Novel objects, *F*(1, 40) = .256, *p* = .616, *η*_*p*_^*2*^ = .006. See Fig 3.

To understand, at a more fine-grained level, the increase in performance between Recognition and Retention found for the SLI group but not the TD group, we tested whether this pattern held separately for Real and Novel items. Note that although the three-way interaction between Group, Real/Novel and Delay was not significant, such higher-level interactions are difficult to obtain, leaving open the possibility that the observed patterns for the SLI and TD groups could differ between Real and Novel items. However, consistent with the lack of a three-way interaction, the SLI group showed an increase in performance between Recognition and Retention for both types of items (Real: *F*(1, 20) = 7.745, *p* = .011, *η*_*p*_^*2*^ = .279; Novel: *F*(1, 20) = 11.65, *p* = .003, *η*_*p*_^*2*^ = .368), whereas the TD group did not show an increase for either (Real: *F*(1, 20) = 0.20, *p* = .889, *η*_*p*_^*2*^ = .001; Novel: *F*(1, 20) = 1.412, *p* = .249, *η*_*p*_^*2*^ = .066). Interestingly, however, the two groups did not differ for either Real or Novel items at either Delay period, with the notable exception of Real items at Recognition, where the TD group showed superior performance (Recognition: Real: *F*(1, 40) = 5.150, *p* = .029, *η*_*p*_^*2*^ = .114; Novel: *F*(1, 40) = 1.349, *p* = .252, *η*_*p*_^*2*^ = .033. Retention: Real: *F*(1, 40) = .724, *p* = .400, *η*_*p*_^*2*^ = .018; Novel: *F*(1, 40) = .000, *p* = .998, *η*_*p*_^*2*^ = .000).

### Recognition and retention of verbal information

Performance on the Verbal subtask is shown in Table 3. Both the SLI and TD groups showed above-chance performance at both Real and Novel items, for both Recognition and Retention, with the exception of the SLI group at Novel items at Recognition (Table 3).

**Table 3:** Recognition and retention accuracy for verbal information.

	SLI	TD
Recognition (10 minute delay)		
Real		
* d’*	0.83 (0.83) ***	1.51 (0.49) ***
Hit Rate	0.71 (0.17)	0.74 (0.09)
False Alarm Rate	0.42 (0.31)	0.22 (0.13)
Novel		
* d’*	0.05 (0.61)	0.91 (0.62) ***
Hit Rate	0.28 (0.17)	0.42 (0.20)
False Alarm Rate	0.27 (0.23)	0.16 (0.14)
Retention (24 hour delay)		
Real		
* d’*	0.65 (0.65) ***	1.14 (0.69) ***
Hit Rate	0.55 (0.27)	0.64 (0.21)
False Alarm Rate	0.35 (0.27)	0.27 (0.21)
Novel		
* d’*	0.32 (0.43) **	1.09 (0.67) ***
Hit Rate	0.33 (0.17)	0.52 (0.23)
False Alarm Rate	0.05 (0.03)	0.06 (0.03)

Note. Accuracy in the verbal subtask, showing means (and standard deviations) of *d*’, as well as of hit rates and false alarm rates. SLI: specific language impairment; TD: typically developing. Asterisks indicate performance greater than chance (mean *d*’s significantly greater than zero, one-sample t-tests, df = 20)

***: p < .001

**: p < .01

*: p < .05.

**Fig 4:**
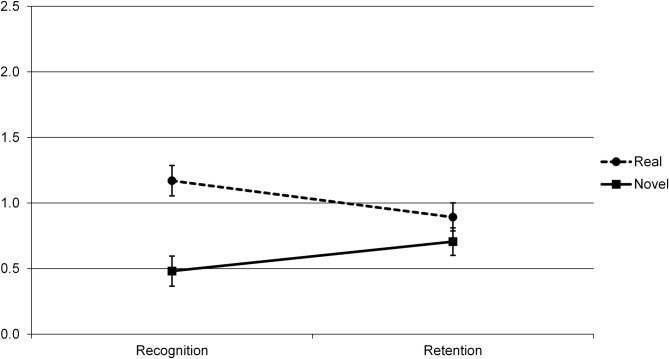
Verbal subtask performance by Delay (10 minutes/Recognition vs. 24 hours/Retention) and Real/Novel, showing mean *d*’ scores and standard errors.

The 2 (Group) X 2 (Real/Novel) X 2 (Delay) ANOVA yielded main effects of Group (*F*(1, 40) = 21.86, *p* = .0001, *η*_*p*_^*2*^ = .353), with better overall performance by the TD children than children with SLI, and Real/Novel (*F*(1, 40) = 24.173, *p* < .0001, *η*_*p*_^*2*^ = .377), with Real words recognized better than Novel ones. There was no main effect of Delay (*F*(1, 40) = .148, *p* = .703, *η*_*p*_^*2*^ = .004). However, the main effect of Real/Novel was qualified by a significant Real/Novel X Delay interaction (*F*(1, 40) = 20.359, *p* < .0001, *η*_*p*_^*2*^ = .337, see Fig 4 below). No other interactions were significant (Group X Real/Novel: *F*(1, 40) = 1.700, *p* = .200, *η*_*p*_^*2*^ = .041; Group X Delay: *F*(1, 40) = .952, *p* = .335, *η*_*p*_^*2*^ = .023; Group X Real/Novel X Delay: *F*(1, 40) = .221, *p* = .641, *η*_*p*_^*2*^ = .005).

To investigate the Real/Novel X Delay interaction, we examined the effect of Delay separately for Novel and Real words, collapsing across the two participant groups. For both types of words there were significant effects of Delay, but in opposite directions for the two types of words. Whereas performance on Novel words increased between Recognition and Retention (*F*(1, 41) = 5.487, *p* = .024, *η*_*p*_^*2*^ = .118), performance on Real words decreased during this interval (*F*(1, 41) = 12.099, *p* = .001, *η*_*p*_^*2*^ = .228). Additionally, the effect of Real/Novel was significant at Recognition (*F*(1, 41) = 38.935, *p* < .0001, *η*_*p*_^*2*^ = .487), but only approached significance at Retention (*F*(1, 41) = 3.480, *p* = .069, *η*_*p*_^*2*^ = .078)–though in both cases performance on Real words was higher than on Novel words. See Fig 4.

### Comparison of performance on the nonverbal and verbal subtasks

To compare overall performance on nonverbal and verbal items directly, we ran a 2 (Group: SLI vs. TD) X 2 (Modality: Nonverbal vs. Verbal) X 2 (Delay: 10 minutes/Recognition vs. 24 hours/Retention) mixed ANOVA on normalized *d*’ scores (for simplicity, averaged over Real and Novel items). This analysis yielded main effects of Modality (F(1,40) = 43.721, p < 0.001, *η*_*p*_^2^ = 0.522), with better performance on the nonverbal than verbal subtask, of Delay (F(1,40) = 7.688, p = 0.008, *η*_*p*_^2^ = 0.161), with better performance at Retention than Recognition, and of Group (F(1,40) = 7.038, p = 0.011, *η*_*p*_^2^ = 0.150), with the TD group showing better performance overall than the children with SLI. However, these main effects were qualified by an interaction between Group and Modality that approached significance (F(1,40) = 3.822, p = 0.058, *η*_*p*_^2^ = 0.087), and significant interactions both between Group and Delay (F(1,40) = 5.426, p = 0.025, *η*_*p*_^2^ = 0.119) and Modality and Delay (F(1,40) = 15.189, p < 0.001, *η*_*p*_^2^ = 0.275). The three-way interaction was not significant (F(1,40) = 2.708, p = 0.108, *η*_*p*_^2^ = 0.063).

Following up on these interactions, we found, first of all, that Modality had a significant effect in both groups, with better performance on the nonverbal than verbal subtask, though this effect was larger in the SLI group (SLI: F(1,20) = 34.636, p < 0.001, *η*_*p*_^2^ = 0.634; TD: F(1,20) = 11.531, p = 0.003, *η*_*p*_^2^ = 0.366). The Group by Delay interaction was explained by better overall performance (over both the Nonverbal and Verbal conditions) at Retention than Recognition by the SLI group but not the TD group (SLI: F(1,20) = 11.348, p = 0.003, *η*_*p*_^2^ = 0.362; TD: F(1,20) = 0.115, p = 0.738, *η*_*p*_^2^ = 0.006). Finally, follow up analyses for the Modality by Delay interaction revealed a significant effect, over both groups, in the nonverbal subtask, with better performance at Retention than Recognition, but not for in the verbal subtask (Objects: F(1,20) = 16.947, p < 0.001, *η*_*p*_^2^ = 292; Words: F(1,20) = 0.654, p = 0.423, *η*_*p*_^2^ = 0.016).

### Incidental encoding

This paper investigates potential SLI/TD differences in learning and retention in declarative memory. Hence our analyses focus on examining Recognition and Retention differences between the two groups. However, each subtask also includes an incidental encoding phase, in which participants had to judge whether the stimulus they saw or heard was real or novel. Performance at the encoding phases of both the Nonverbal and Verbal subtasks is shown in Table 4. Analyses were run with normalized *d’* as the dependent measure. Both the SLI and TD groups showed above-chance performance at both subtasks (Table 4).

**Table 4:** Encoding accuracy in the Nonverbal and Verbal subtasks.

	SLI	TD
Nonverbal		
*d’*	1.99 (1.13) ***	2.65 (0.80) ***
Hit Rate	0.87 (0.14)	0.91 (0.08)
False Alarm Rate	0.27 (0.24)	0.16 (0.13)
Verbal		
*d’*	2.43 (0.74) ***	3.49 (0.54) ***
Hit Rate	0.87 (0.06)	0.93 (0.05)
False Alarm Rate	0.14 (0.13)	0.03 (0.04)

Note. Accuracy in the Encoding phase, showing means (and standard deviations) of *d*’, as well as of hit rates and false alarm rates. SLI: specific language impairment; TD: typically developing. Asterisks indicate performance greater than chance (mean *d*’s significantly greater than zero, one-sample t-tests, df = 20):

***: p < .001.

To examine potential group differences in incidental encoding we performed separate one-way ANOVAs on the Nonverbal and Verbal subtasks. These revealed group differences in both subtasks, with the TD group showing significantly better performance than the SLI group in categorization accuracy (i.e., Real vs. Novel) for both Nonverbal and Verbal stimuli (Nonverbal: *F*(1, 40) = 4.789, *p* = .035, *η*_*p*_^*2*^ = .107; Verbal: *F*(1, 40) = 28.273, *p* < .001, *η*_*p*_^*2*^ = .414); see Table 4.

These group differences in incidental encoding could potentially affect the group differences reported above for Recognition and Retention. Note that because the encoding was incidental, there is no clear direct relation between success at this phase (distinguishing Real and Novel items) and actually learning the material, which is tested later in Recognition and Retention. Nevertheless, we ran ANCOVAs parallel to the ANOVAs presented above for both the Nonverbal and Verbal subtasks, with encoding (normalized *d’*) included as a covariate.

Importantly, the same pattern of critical results was obtained as for the ANOVAs presented above. The ANCOVA for the Nonverbal task crucially yielded an interaction between Group and Delay (*F*(1, 39) = 7.068, *p* = .011, *η*_*p*_^*2*^ = .153; all other effects: *p*s > .3). Likewise, the ANCOVA for the Verbal subtask yielded a similar pattern of results as the ANOVA presented above for this subtask, namely only a borderline significant interaction between Real/Novel and Delay (*F*(1, 39) = 3.833, *p* = .057, *η*_*p*_^*2*^ = .089), with no other significant effects (*p*s > .1).

## Discussion

The purpose of this study was to examine both learning and retention in declarative memory in SLI, of both nonverbal and verbal information. In order to obtain a clear picture of the status of declarative memory, the study attempted to minimize the influence of functions that can affect measures of learning and retention in this system and are often impaired in SLI, namely free recall and short-term memory. We aimed to achieve this goal by probing declarative memory with a recognition memory task, following incidental encoding. To test for learning we examined recognition memory 10 minutes after encoding, separately for nonverbal items (real and novel objects) and verbal items (real and novel words). To test for retention of this information in the same individuals, we then examined recognition memory of the same items 24 hours later, allowing us to investigate potential differences in overnight retention between the SLI and TD groups. To our knowledge, this is the first study to test incidental learning in declarative memory in SLI, and the second (after McGregor et al., 2013) to test for overnight retention in this system in the disorder.

Analyses revealed the following pattern. In the nonverbal domain we found a Group (SLI vs. TD) by Delay (10 minutes vs. 24 hours) interaction. Analyses revealed that the children with SLI improved significantly in their recognition memory between testing for learning (at the short delay, that is, after 10 minutes) and testing for retention one day later, whereas the TD children showed no change in performance during this period. This pattern held for both real and novel objects. Interestingly, the two groups did not differ significantly in their recognition memory for either real or novel objects at either delay period, except for real objects after the short delay, when the children with SLI performed worse than the TD children.

In the verbal domain, a main effect of group showed that the TD children performed better overall at recognition memory than the children with SLI, that is, over both real and novel words, over both delay periods. Additionally, analyses revealed an improvement of recognition memory for novel words between the short delay and one day later, over both groups. In contrast, recognition memory for real words decreased during this time period over the two groups. Unlike in the nonverbal domain, no interaction between Group and Delay was found.

An analysis directly comparing performance between the nonverbal and verbal subtasks revealed a Group by Delay interaction over both subtasks, due to better overall performance at the long than short delays only in the SLI group, with no three-way Group by Modality by Delay interaction. Additionally, this analysis revealed overall better performance (over both delay periods) at the nonverbal than verbal items in both groups, with this difference being larger in the SLI group.

The results suggest the following patterns regarding declarative memory in SLI, at least when tested with a recognition memory paradigm with incidental encoding, with recognition tested minutes after encoding and then again one day later. In the nonverbal domain, children with SLI appear to have recognition memory deficits only for real objects, and only at a short delay of minutes. Importantly, they do not show recognition memory deficits for real objects one day after learning the items, and do not show impairments for novel objects at either delay period. Moreover, only children with SLI *improve* at remembering items between initial learning and testing one day later, and in fact do so for both real and novel items. In contrast, TD children show no changes in performance during this period. In the verbal domain, children with SLI appear to have recognition memory deficits for both real and novel words, at both short and long delays. However, the *changes* in performance between the short and long delays do not differ between the groups for verbal items.

A key question is how the observed patterns may best be interpreted. First of all, it does not seem likely that these findings can be accounted for by differences between the TD and SLI groups at the incidental encoding task. Success at distinguishing real and novel items in this task does not have any clear relation with actually encoding the material. Moreover, ANCOVAs with performance from the encoding task covaried out yielded similar patterns to those from the ANOVAs without this factor included.

Second, it might be argued that the improvements between the two delay periods observed in the nonverbal task for the SLI but not the TD group could be due to ceiling effects for the latter. On this view, the lack of an increase between the two delay periods for the TD group might simply be explained by the fact that their performance was already very good at the nonverbal task after the short delay, and hence they had less room for improvement. Indeed, the highest performance at the short delay was observed for the TD group, for real objects, with an accuracy score (over hits and correct rejections) of 82%. However, the TD group’s accuracy for novel objects was only 61%, yet they showed no improvements for either novel or real objects. In contrast, the SLI group showed improvements at both real and novel objects, even though their accuracy at real objects was higher at the short delay (72%) than it was for the TD group for novel ones (i.e., 61%). Moreover, even for real objects, accuracy for the TD group at the short delay (i.e., 82%) was not particularly close to ceiling (i.e., 100%). Indeed, the variance of *d*-prime scores for real objects at the short delay did not differ between the TD and SLI groups, also arguing against ceiling effects for the TD group for this condition (see Table 2; Levene’s test for equality of variance: *F*(1, 40) = .319; *p* = .575). Together, the data suggest that ceiling effects are unlikely to explain the pattern of improvements at the nonverbal task between the two delays for the SLI but not the TD group.

Third, it might be suggested that the improvements observed between the short and long delays for the SLI but not the TD group could be explained by worse initial learning by the children with SLI. In particular, since the children with SLI showed worse performance than the TD children at real objects at the short delay, it could be argued that they would be positioned to show more additional learning with an additional exposure (i.e., of the target items during the recognition memory task at the short delay)–assuming a classic non-linear (e.g., log-shaped) learning curve [48,49], since the performance of the children with SLI at the short delay is “further left” on the curve. On this view, such additional learning could result in greater improvements in the SLI than TD group between the short and long delay. Alternatively, it might be argued that the children with SLI in particular understood the instructions better the second time, at the long delay. In either case, however, the children with SLI were not significantly worse than the TD children on *novel* objects at the short delay; yet the same pattern was observed on these items as on the real objects, namely, an improvement between the two delays for the children with SLI but not the TD children. Additionally, the performance at the short delay was lower for *both* participant groups on novel objects than even the SLI group on real objects (see Table 2), yet only the SLI group showed improved performance between the delays, moreover on *both* real *and* novel objects. Overall, this suggests that lower performance at the short delay is unlikely to account for the increases between the delays observed for the SLI group but not the TD group.

We suggest instead that the group differences observed in the changes between testing for initial learning and for retention 24 hours later for both real and novel items in the nonverbal task could be due to group differences in *consolidation*. Consolidation, as we have seen above, refers to the stabilization of memories after their initial acquisition. This process, which depends on the medial temporal lobes as well as neocortical regions [11,50,51], and whose molecular mechanisms are quite well studied [52–54], has been examined not only extensively in non-human animals, but also in humans. In humans, consolidation has been observed for both verbal and nonverbal information, over various time periods, ranging from hours to days to weeks [55–58]. Studies have revealed the importance of sleep in consolidation, showing that sleep can help preserve information, often with better retrieval of the learned information after a period involving sleep than after the same period without sleep [56,59–62]. Some of these studies show that sleep can lead to enhanced retrieval not only as compared to conditions without sleep, but even as compared to initial learning [61,62].

Based on the results from the present study, we suggest that the children with SLI may show consolidation strengths in declarative memory, as compared to TD children, at least for nonverbal items over the course of 24 hours with sleep. These strengths seem to hold for different types of nonverbal items, given that increases between the two delays were found for children with SLI but not TD children for both real and novel nonverbal items. Indeed, these strengths seem to lead to normal recognition performance in children with SLI after 24 hours even for items that showed impaired performance at initial learning (i.e., real objects, at the short delay).

The lower recognition memory performance of the SLI than TD group at real (but not novel) objects at the short delay might be explained by the fact that these items are associated with verbal labels, which could impair their processing. This would not be surprising, given the language difficulties found in children with SLI, including with phonology. It is also consistent with the particular impairment observed in this study for the SLI group at the verbal task, including at encoding. More generally, the findings strengthen the view that declarative memory problems in SLI in the verbal domain, in particular with word learning, may be due primarily to language problems rather than to declarative memory per se [2,6].

Although the SLI group was worse than the TD group at verbal items at both the short and long delays, the *change* in performance between the delays did not differ between the groups. This suggests that although consolidation was not enhanced in the SLI group for verbal items, neither was it impaired; rather, the children with SLI showed evidence for normal consolidation in the verbal domain. However, various questions remain about SLI and TD consolidation of verbal items. First, future studies may elucidate why, over both groups, there was a decrease between the two delays for real words, but an increase for novel words. Second, potential group differences between the two groups in consolidation may also be revealed by further research. Although in the current study there was no interaction between group and delay for the verbal material, exploratory analyses on each group, carried out separately for real and novel words as was done for the nonverbal material, suggested an intriguing pattern. For real words, although both groups showed signs of a decrease in performance between the short and long delay, this reached significance only for the TD group (SLI: *F*(1, 20) = 3.035, *p* = .097, *η*_*p*_^2^ = .132; TD: *F*(1, 20) = 9.787, *p* = .005, *η*_*p*_^2^ = .329). Moreover, for novel words, although both groups showed increases between the two delays, this effect reached borderline significance for the SLI group (*F*(1, 20) = 4.314, *p* = .051, *η*_*p*_^*2*^ = .177) but not for the TD group (*F*(1, 20) = 1.591, *p* = .222, *η*_*p*_^*2*^ = .074). These exploratory analyses suggest that TD but not SLI children might show a decrement in performance for real words between the two delays, while only the children with SLI show an improvement at novel words, hinting at the possibility of SLI consolidation strengths in the verbal domain as well. The Group by Delay interaction yielded by the analyses comparing performance on the nonverbal and verbal subtasks, due to better overall performance, only in the SLI group, at the long than short delays over *both* subtasks, with no three-way Group by Modality by Delay interaction, further supports the possibility of SLI consolidation strengths in the verbal domain. Future studies focusing on this issue, with large sample sizes and other tasks, may be useful.

The factors and mechanisms underlying the apparent SLI strengths at consolidation in declarative memory remain to be elucidated. One obvious possibility, though still speculative, is that they may be related in some way to sleep, since previous evidence suggests that sleep, in particular Slow Wave Sleep, is especially important for consolidation in this system [60,63–65]. Indeed, as discussed above, sleep has been found to lead to *improvements* at remembering items as compared to initial learning, as was observed in the present study. Perhaps children with SLI spend more time in Slow Wave Sleep, or have more efficient sleep-related consolidation processes for declarative memory, as compared to TD children. However, further research is required before sleep-related factors can be identified as a source of the observed patterns. Alternatively or in addition, declarative memory consolidation advantages in SLI might be related to the “seesaw” effect, that is, to the enhancement of declarative memory due to impairments of procedural memory [5,12]. On this view, as suggested by the Procedural Deficit Hypothesis (PDH) and the broader Declarative/Procedural model framework upon which the PDH is built (see below), the procedural memory impairments in SLI that appear to lead to their grammatical (and other) deficits may be associated with improvements of declarative memory, due to the seesaw effect. Such an interaction between memory systems might be expected particularly in developmental disorders, given the continuing interactions between systems during development [66,67]. Although the mechanisms of the seesaw effect remain to be elucidated [5,12], and could indeed be related to sleep, the present findings suggest that at least one manifestation of these effects might be related to consolidation, rather than (just) initial learning. Note that no seesaw effect was reported in children with SLI in Kuppuraj et al. [25]; however, this does not counter the possibility of seesaw effects in consolidation in SLI, since in that study retention was examined only one hour after encoding, at which point strong consolidation effects might not be expected.

### Implications and future directions

Although this is the first study to suggest possible SLI strengths in declarative memory, and further research and confirmation is clearly needed, the study has various potential implications, and opens up new avenues of research.

First of all, the results support and suggest refinements of the PDH of SLI, specifically regarding the status of declarative memory. In addition to positing abnormalities of brain structures underlying procedural memory, the PDH hypothesizes that in individuals with SLI declarative memory should be largely spared, particularly for nonverbal information, and may even show advantages compared to TD individuals, due to the seesaw effect [5–7]. The findings from the present study are consistent with these predictions, and refine them by revealing not only the normal attainment of nonverbal knowledge in SLI, but, for the first time, apparent retention strengths in declarative memory, which may be related to consolidation.

Additionally, the results suggest future areas of research for the Declarative/Procedural (DP) model, on which the PDH is based. In particular, the possibility of consolidation strengths in declarative memory in SLI, together with evidence suggesting consolidation impairments in procedural memory in SLI [17], suggest that dissociations between lexical/declarative memory and grammatical/procedural processes may extend to consolidation. Future studies should thus further investigate consolidation in the two memory systems and how these might affect language [68].

Apparently normal (or possibly enhanced) consolidation in the verbal domain in SLI might help explain the relative sparing of lexical knowledge in children with the disorder, compared to aspects of grammar [1,6], since learning of lexical but not grammatical knowledge seems to rely critically on declarative memory [5,12]. It could also at least partly explain the observation that lexical abilities appear to gradually improve as children with SLI get older [69,70], in particular because declarative memory improves during childhood [12]. Note that a dependence of lexical memory on declarative memory does not preclude an additional reliance of lexical memory on, or interactions with, various functions impaired in SLI, such as phonology, syntax, working memory, or recall, which would be expected to lead to some level of lexical deficits, perhaps continuing throughout the lifespan [6]. Additionally, note that normal or enhanced consolidation in declarative memory is consistent with and may help explain why this memory system seems to play a compensatory role for grammar in children with SLI (5–7).

The findings reveal, for the first time, the possibility that children with SLI show cognitive *strengths*, as compared to typically developing children. Strengths in various domains and functions have been observed for a variety of disorders, including in declarative memory in both dyslexia and autism [7,71–73]. However, to our knowledge cognitive strengths have never been reported for SLI, in any domain. The findings presented here suggest that children with SLI also show such strengths, perhaps in consolidation in declarative memory. Further studies examining this issue seem warranted.

Possible strengths in declarative memory in SLI are also consistent with the compensation underdiagnosis hypothesis [7]. On this view, SLI may be underdiagnosed partly as a result of compensation by declarative memory, in particular for grammatical/procedural deficits. If indeed aspects of declarative memory are enhanced in SLI, including possibly in the verbal domain (see above), this would facilitate compensation, potentially increasing underdiagnosis of the disorder.

The findings suggest the need for further investigation of declarative memory consolidation in SLI. This could elucidate how broadly the apparent consolidation strengths in this system may hold in SLI–for example, across age groups in both children and adults, types of items (e.g., other types of nonverbal knowledge, such as faces or scenes), tasks (including those that are less episodic in nature, as well those that involve free or cued recall), and delay periods, and how these may interact with sleep and the time of day during which encoding occurs [33]. (Note that comparisons between the present study and McGregor et al. (2013) are difficult, given the difficulty in interpreting results from that study, and the differences between the studies, including incidental vs. intentional training, testing with recognition vs. recall, and the nature of the participants; see Introduction). Of particular interest, it remains to be seen whether children with SLI might show *better* memory than TD children for nonverbal (and perhaps verbal) information after *longer* periods, during which additional consolidation could take place. The apparent procedural memory consolidation deficits in SLI [17] should also be further examined, including the underlying mechanisms. Overall, such investigations of consolidation in SLI may elucidate not only the nature of SLI, but also of consolidation more generally.

The particular patterns of performance of hits and false alarms in the present study also warrant further investigation. This pattern suggests that the SLI consolidation strengths might be due to a larger reduction of false alarms between Recognition and Retention in the SLI than the TD group, rather than an increase in the number of hits, at least in the case of real objects (see Table 2), and perhaps also for novel words (see Table 3). Although research is sparse on hits versus false alarms in retention and consolidation, there is some evidence in the literature that in recognition memory tasks, sleep reduces false recognition (false alarms), while it does not affect correct recognition (hits) [74], consistent with the pattern observed here. Alternatively, it might be argued that participants with SLI may have better understood the requirements of the recognition memory task the second time (i.e., at Retention), perhaps leading to a more consistent rejection of foils, i.e. to a lower number of false alarms. On this account, however, it is unclear why an SLI reduction in false alarms from Recognition to Retention would be found for real but not novel objects (see Table 2), and perhaps novel but not real words (see Table 3). Future studies, with larger numbers of participants, might clarify these issues. Additionally, it remains to be seen why the impaired SLI performance on some conditions (e.g., real objects and real words at the short delay), as compared to TD children, seems to be primarily due to differences in false alarms rather than in hits (see Tables 2 and 3).

The findings of the present study suggest that retention in declarative memory should also be further examined in other disorders. It should be investigated particularly in disorders that may be related to SLI, as evidenced by comorbidities with SLI and similar patterns of deficits and spared functions, including of declarative memory [7,75]. These may include dyslexia, autism, Tourette syndrome, obsessive-compulsive disorder, and ADHD [7]. Indeed, one study found enhanced declarative memory in dyslexia, although the superior performance was observed across both short and long (one day) delays, with no improvement between them [72]. Future studies on consolidation in such disorders seem warranted.

The results of the present study also have methodological implications. In particular, the study suggests that examining the status of declarative memory may be usefully carried out with tasks that minimize the involvement of other functions that interact with, but are not necessary for, the functioning of this system, such as free recall and working memory. This approach seems particularly important in the many developmental and other disorders where these functions are problematic [7]. More generally, examining the status of a neurocognitive function may be best carried out by tasks that minimize other, non-critical, functions, especially if these may be impaired.

Finally, the study of course has limitations, which could be addressed by future research. For example, future studies could attempt to match the TD and SLI groups on performance of real objects at the short delay, in order to test whether group differences in consolidation would still be found, even for real objects. Additionally, whereas in this study the same target items were presented at both the short and long delays, future studies could include different target items in the different test sessions, thereby avoiding potential problems of group learning differences, as discussed above.

## Conclusion

In conclusion, the present study reveals normal and perhaps even enhanced consolidation in declarative memory in SLI. To our knowledge this is the first demonstration of apparent cognitive strengths in children with SLI. The findings, should they be supported by further studies, have a range of basic research and clinical implications for SLI as well as for related disorders, and open up new avenues of research.

## Supporting Information

S1 Fig **Individual participant performance on the nonverbal task for the SLI (A) and TD (B) groups.** The figures show *d*’ performance for each individual, collapsed over both Real and Novel items, at both Recognition (10 minutes after encoding) and Retention (24 hours after encoding). SLI: children with specific language impairment; TD: typically-developing children; *d*': d-prime scores.
(DOCX)

S1 Dataset Dataset behind analyses in ‘Learning and overnight retention in declarative memory in specific language impairment’. (XLS)
