# Supplementary material for: Learning and Overnight Retention in Declarative Memory in Specific Language Impairment
Source: PLoS One. 2017 Jan 3;12(1):e0169474. doi: 10.1371/journal.pone.0169474 (PMC5207735; doi:10.1371/journal.pone.0169474)
Supplement: S1 Fig — Individual participant performance on the nonverbal task for the SLI (A) and TD (B) groups. The figures show d’ performance for each individual, collapsed over both Real and Novel items, at both Recognition (10 minutes after encoding) and Retention (24 hours after encoding). SLI: children with specific language impairment; TD: typically-developing children; d': d-prime scores. (DOCX) [file pone.0169474.s001.docx]

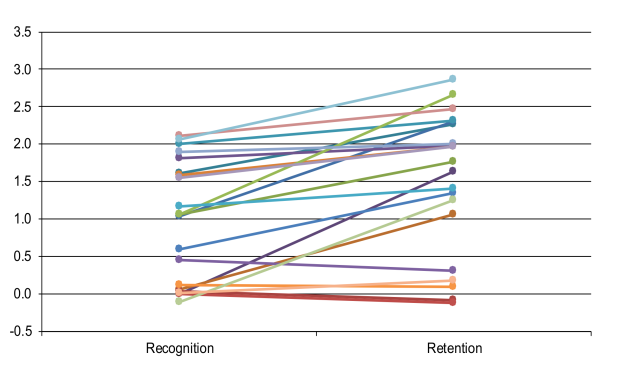

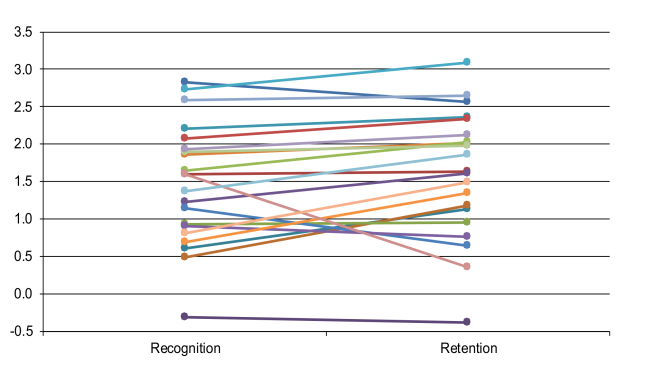


B. TD ttTD?TTddddd.

A. SLI

**S1 Fig. Individual participant performance on the nonverbal task for the SLI (A) and TD (B) groups.** The figures show *d*’ performance for each individual, collapsed over both Real and Novel items, at both Recognition (10 minutes after encoding) and Retention (24 hours after encoding). SLI: children with specific language impairment; TD: typically-developing children; *d*': d-prime scores.
